# Mobility and kinship in the world’s first village societies

**DOI:** 10.1073/pnas.2209480119

**Published:** 2023-01-17

**Authors:** Jessica Pearson, Jane Evans, Angela Lamb, Douglas Baird, Ian Hodder, Arkadiusz Marciniak, Clark Spencer Larsen, Christopher J. Knüsel, Scott D. Haddow, Marin A. Pilloud, Amy Bogaard, Andrew Fairbairn, Jo-Hannah Plug, Camilla Mazzucato, Gökhan Mustafaoğlu, Michal Feldman, Mehmet Somel, Eva Fernández-Domínguez

**Affiliations:** ^a^Department of Archaeology, Classics and Egyptology, University of Liverpool, Liverpool L69 7WZ, United Kingdom; ^b^National Environmental Isotope Facility, British Geological Survey, Nottingham NG12 5GG, United Kingdom; ^c^Archaeology Center, Department of Anthropology, Stanford University, Palo Alto, CA 94305; ^d^Faculty of Archaeology, Adam Mickiewicz University, 61-614 Poznań, Poland; ^e^Department of Anthropology, The Ohio State University, Columbus, OH 43210; ^f^UMR-5199 De la Préhistorie á L’Actuel: Culture, Environnement, et Anthropologie (PACEA), Université de Bordeaux, Pessac Cedex, 33615 France; ^g^Department of Cross-Cultural and Regional Studies, University of Copenhagen, 2300 Copenhagen S, Denmark; ^h^Department of Anthropology, University of Nevada, Reno, NV 89557; ^i^Institute of Archaeology, University of Oxford, Oxford OX1 2PG, United Kingdom; ^j^Santa Fe Institute, Santa Fe, NM 87501; ^k^School of Social Science, The University of Queensland, Brisbane, QLD 4072, Australia; ^l^Department of Archaeology, Faculty of Letters, Ankara Hacı Bayram Veli University, Yenimahalle, 06570 Ankara, Turkey; ^m^Archaeo- and Palaeogenetics Group, Institute for Archaeological Sciences, University of Tübingen, 72074 Tübingen, Germany; ^n^Senckenberg Centre for Human Evolution and Palaeoenvironment, University of Tübingen, 72074 Tübingen, Germany; ^o^Department of Biological Sciences: Biology/Molecular Biology and Genetics, Middle East Technical University, 06800 Ankara, Turkey; ^p^Department of Archaeology, University of Durham, Durham DH1 3LE, United Kingdom

**Keywords:** stable isotopes, kinship, early villages

## Abstract

Around 10,000 y ago in southwest Asia, the cessation of a mobile lifestyle and the emergence of the first village communities during the Neolithic marked a fundamental change in human history. The first communities were small (tens to hundreds of individuals) but remained semisedentary. So-called megasites appeared soon after, occupied by thousands of more sedentary inhabitants. Accompanying this shift, the material culture and ancient ecological data indicate profound changes in economic and social behavior. A shift from residential to logistical mobility and increasing population size are clear and can be explained by either changes in fertility and/or aggregation of local groups. However, as sedentism increased, small early communities likely risked inbreeding without maintaining or establishing exogamous relationships typical of hunter-gatherers. Megasites, where large populations would have made endogamy sustainable, could have avoided this risk. To examine the role of kinship practices in the rise of megasites, we measured strontium and oxygen isotopes in tooth enamel from 99 individuals buried at Pınarbaşı, Boncuklu, and Çatalhöyük (Turkey) over 7,000 y. These sites are geographically proximate and, critically, span both early sedentary behaviors (Pınarbaşı and Boncuklu) and the rise of a local megasite (Çatalhöyük). Our data are consistent with the presence of only local individuals at Pınarbaşı and Boncuklu, whereas at Çatalhöyük, several nonlocals are present. The Çatalhöyük data stand in contrast to other megasites where bioarchaeological evidence has pointed to strict endogamy. These different kinship behaviors suggest that megasites may have arisen by employing unique, community-specific kinship practices.

## Significance

Strontium and oxygen isotopes were measured for adults who lived in southwest Asia during the foraging-to-farming transition. Data spanning seven millennia show limited mobility during the early Holocene and local partner exchange within small hunter-gatherer communities in the late Pleistocene. Conversely, later megasites show more mixed patterns of mobility and kinship, with greater genetic diversity and more nonlocals immigrating to these sites. We argue that these data show that the key agents in local kinship practices prior to the emergence of farming were derived more from shared ideologies and associations involving fictive kin (e.g., neither consanguineous [blood] nor affinial [marriage-like] ties). Continuity and diversity in kinship practices suggest that the world’s first villages included unique social and biological kinship identities.

**Fig. 1.**
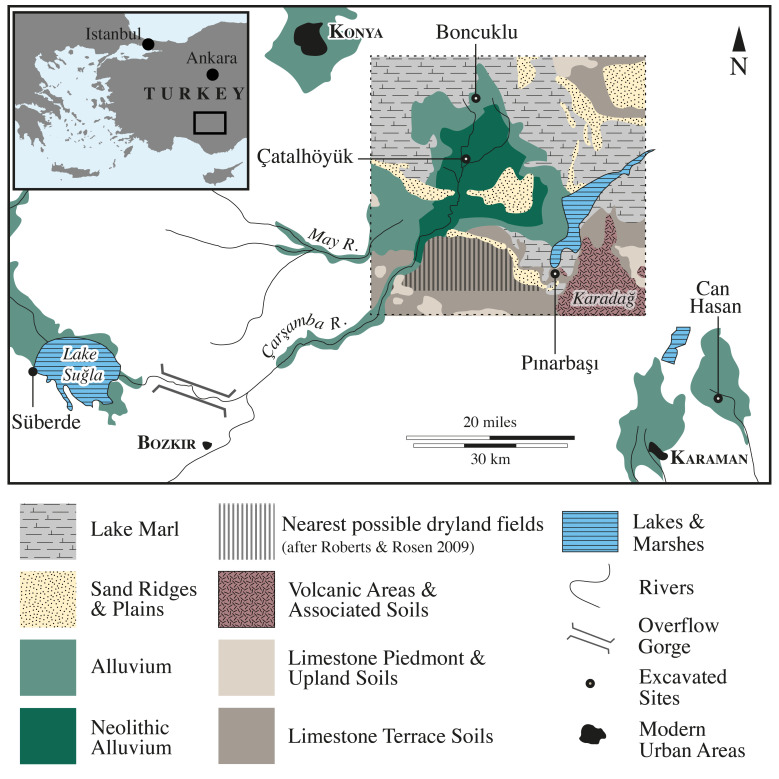
The Konya paleolake basin detailing the geological area in which the sites are situated. After Roberts and Rosen (13) and de Meester (14).

During the terminal Pleistocene and early Holocene the first human communities gradually reduced their mobile lifestyle and began to establish the world’s first villages. Archaeological evidence reveals small villages measuring only a few hectares at most that likely supported tens to hundreds of people (1–6). From around 7500 cal BC larger sites began to emerge, including some that were exceptionally so by both earlier and contemporary standards. These sites measured more than 10 ha, supported thousands of people, and have been described as “megasites” (3, 4, 7). Much attention has been focused on the first villages and the subsequent emergence of megasites since they signal fundamental shifts in human behavior. An increasing emphasis was placed on logistical versus residential mobility, there was change and continuity in both architecture and material culture traditions, distinctions in social identities, a florescence in ritual behavior, and the gradual replacement of wild food resources initially with managed and later domesticated ones, although it should be noted that many practices often associated primarily with hunter-gatherer societies, such as mobility and hunting, also persisted and did not develop side by side in a linear fashion with other shifts (6). These changes were accompanied by, and inextricably linked to, the occupation of sites by long-term, coresident groups and exponential population increase (8). However, the kinship mechanisms that enabled Neolithic populations to develop from small communities to megasites have not been thoroughly explored. Here, we test the hypothesis that early sedentarizing communities would have risked close inbreeding without maintaining or establishing exogamous relationships typical of mobile hunter-gatherers (9, 10). In contrast, larger populations associated with megasites made endogamy sustainable with limited risk. To document and interpret the role of endogamous versus exogamous kinship behaviors in the origins of village life and the rise of megasites, we measured strontium and oxygen isotopes in tooth enamel from 99 individuals from 14th to 12th millennium cal BC Epipaleolithic hunter-gatherer (11) and 10th to 9th millennium cal BC Pınarbaşı (6), 9th to 8th millennium cal BC Early Neolithic Boncuklu (6), and 8th to 7th millennium cal BC later Neolithic megasite of Çatalhöyük (12). See Fig. 1 and *SI Appendix*, *SI Text 1* for site details.

Numerous excavations across southwest Asia have demonstrated that, between the Epipaleolithic and Neolithic, hunter-gatherers of the early Holocene became increasingly sedentary as they constructed more substantial, long-lived dwellings that together formed early village settlements, which endured for hundreds if not thousands of years as fixed places in the landscape (1–6, 15). These dwellings were regularly repaired, renovated, and reconstituted and contain archeobotanical and archeozoological remains attesting to multiseason occupation. This evidence indicates that communities substantially reduced their residential mobility across southwest Asia following the Epipaleolithic. These villages also increased in size during the early Neolithic, with the first megasites appearing in the eighth millennium cal BC during the late Pre-Pottery Neolithic B (PPNB) in the Levant (4). The timing of this exponential population increase, known as the Neolithic Demographic Transition, raises two important questions about the emergence of the first villages. First, how did the earliest villages maintain their semisedentary behavior without risking close inbreeding? Second, were megasites composed largely of endogamous groups of local people who caused exponential population growth from their site-based kinship practices and increasing fertility (4, 16)? Alternatively, did nearby inhabitants simply join forces forming megasites through local and/or regional community aggregation (17, 18)? We hypothesize that close inbreeding was avoided in early, small communities through residential mobility and sociopolitical partner-exchange kinship practices, which effectively mixed local and nonlocal partners. By contrast, risks of within-community mating would have been less severe in the larger populations of megasites, which instead supported endogamous reproduction and enabled these sites to be populated by long-term residents.

## Bioarchaeological Context of Neolithic Southwest Asian Kinship Practices

Archaeological and paleodemographic studies have shown both site size and population density increased dramatically during the eighth millennium cal BC (4, 8) alongside increased fertility (19) and changes to house architecture that are argued to relate to changing social behaviors from the impact of scalar stress and need for more storage (4). These houses, beneath which the dead are most often buried, have been suggested as having been used by families (20) or more complex socially organized kin (21, 22). Bioarchaeology in southwest Asia has since provided an insight into genetic relatedness, enabling past kinship behaviors to be determined directly from human remains via biological anthropology and geochemistry (23–27) and most recently by ancient DNA (28–33); see also *SI Appendix*, *SI Text 2* for an extended discussion. Genetic data, however, provide evidence of the population origins of a community, rather than their birth location. Establishing whether individuals originated from locales where they were buried is critical since high genetic diversity might indicate an influx of nonlocal individuals or specific partner-exchange kinship practices. Previous attempts to address these questions in southwest Asia have lacked at least one of the following: good organic preservation, large sample size, extensive time depth spanning the period of increasing sedentism within or across proximate sites, and extensive field surveys mapping site size and their distribution over time (4, 18, 34). In Anatolia the Çatalhöyük megasite and the nearby early sedentary sites of Boncuklu and Pınarbaşı, which together were occupied during the 14th millennium to 6th millennium cal BC, are all located in the same geologically homogenous region, and are situated 10 to 30 km apart. Genetically these communities have been shown to have arisen largely in situ, with limited gene flow involving populations in the Zagros and Levant. This offers a unique opportunity to identify residential mobility in the emergence of early village society and to establish the role of partner-exchange kinship practices. Evaluating the evidence for practices such as endogamy and exogamy in the rise of megasites and how these compare in smaller, earlier, nearby communities is crucial to understand social and biological kinship practices during a major transition in human population growth with eventual worldwide impact.

## Results

### Strontium Isotope Analysis.

**Fig. 2.**
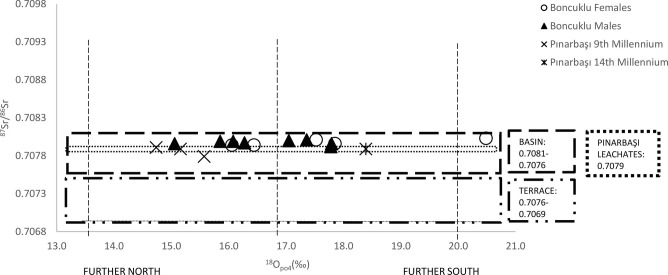
Sr and O isotope values for Boncuklu (*n* = 18) and Pınarbaşı (*n* = 4). Paleolake basin and terrace Sr value constraints follow Bogaard et al. (39). For raw data see *SI Appendix*, Table S3. British Geological Survey data © UKRI 2022.

**Fig. 3.**
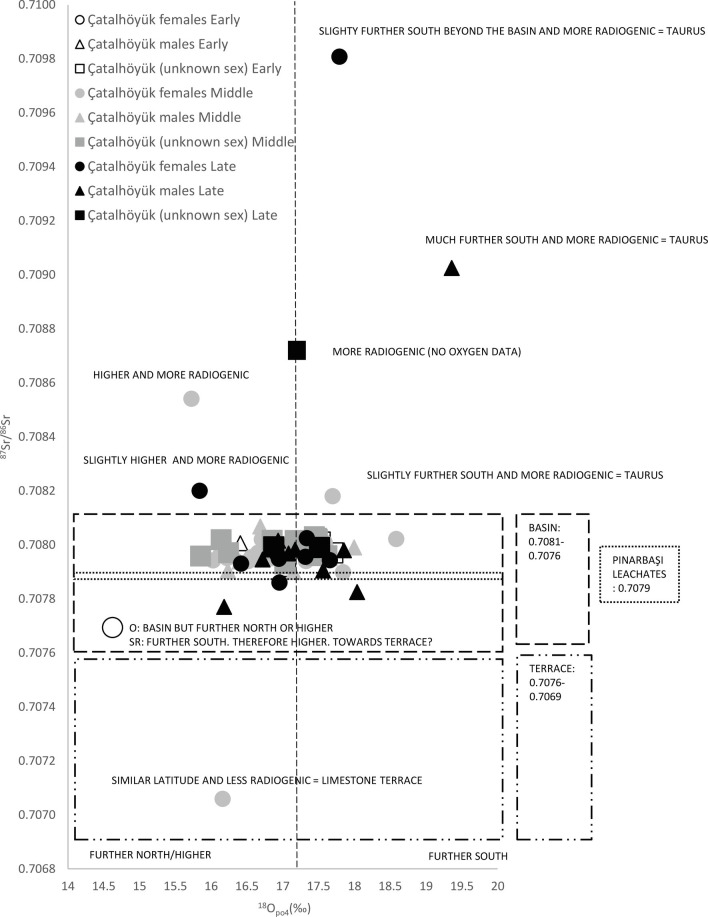
Strontium and oxygen isotope values from Çatalhöyük (*n* = 77) plotted according to site, sex, and occupation period. Paleolake basin and terrace Sr value constraints follow Bogaard et al. (39). For raw data see *SI Appendix*, Table S3. British Geological Survey data © UKRI 2022.

A total of 92 out of the 99 individuals we measured fall within the paleolake basin soils range (Figs. 2 and 3) where our sites are located and thus are consistent with an early childhood spent in this area (see also *SI Appendix*, *SI Text 3* and Fig. 1). Several nonlocal individuals (those beyond the paleolake basin soil range) were found at Çatalhöyük, while at Boncuklu and Epipaleolithic and 10th to 9th millennium cal BC Pınarbaşı these data were consistent with all individuals deriving from the paleolake basin. The nonlocal individuals at Çatalhöyük have ^87^Sr/^86^Sr values consistent with a childhood spent either in, or occasionally traveling to, the Taurus Mountains (higher radiogenic zone) or the limestone terrace (lower radiogenic zone) or originating from an intermediate settlement. At Pınarbaşı, which is the closest to the limestone terraces, the data from the 10th to 9th millennium cal BC and Epipaleolithic periods of occupation are consistent with childhoods spent in the paleolake basin rather than the terraces.

### Oxygen Isotope Analysis.

The mean δ^18^O values for both Boncuklu (δ^18^O_c_ 25.8‰, δ^18^O_p_ 16.9‰) and Çatalhöyük (δ^18^O_c_ 25.9‰, δ^18^O_p_ 17.0‰) individuals are virtually identical and fall within the annual winter-to-summer range for central Turkey. These data are consistent with inhabitants at both sites spending their childhood in the same approximate area (excluding the outlying Sr isotope individuals). There is some overlap between the individuals from Boncuklu and Pınarbaşı which is consistent with a childhood spent in a more northerly region of the paleolake basin and may indicate seasonal wholesale movement. At Boncuklu males and females are similar, although one female has oxygen isotope values consistent with a childhood spent further south in the paleolake basin. Significantly, one individual falling within the interquartile range for the Boncuklu inhabitants also overlaps with the inhabitants from 10th to 9th millennium cal BC Pınarbaşı, which are consistent with a childhood spent in a more northerly region of the paleolake basin and might indicate seasonal wholesale movement by the inhabitants. The data for the Epipaleolithic Pınarbaşı individual is consistent with a childhood spent in the paleolake basin but likely further south than the occupants of Pınarbaşı did later during the 10th to 9th millennium cal BC. At Çatalhöyük those with strontium isotope values consistent with greater mobility or a non/local origin have oxygen isotope values consistent with mobility from a zone with similar latitude and/or altitude to the paleolake. See *SI Appendix*, *SI Text 4* for a more detailed discussion.

### Archaeological Contexts of the Nonlocal and More Mobile Individuals.

The nonlocal/more mobile individuals include both sexes and range in age from adolescent to older adult (50 y or more). See *Materials and Methods* for sample population demographics. At Çatalhöyük, there is nothing to otherwise identify or to distinguish between them individually or as a group. There are no grave goods or personal ornaments, and the buildings they were buried beneath are not particularly elaborate, large, or part of long-lived house sequences. There is also no evidence these individuals were disproportionately afforded secondary burial practices such as cranial or skull retrieval. A tooth from a curated cranium was sampled (sk. 20830) and this individual falls within the range for the paleolake basin. With respect to burial location, the nonlocal individuals are also scattered across houses, but always singly (*SI Appendix*, *SI Text 4* and Figs. S1 and S2). The presence of nonlocal adolescents at Çatalhöyük indicates that some individuals arrived at the site relatively early in life. At Boncuklu, the single possible nonlocal individual (ZHB) was a young adult female who was buried with several perforated shells and other beads. However, other individuals at Boncuklu were also afforded such grave goods. Thus, funerary evidence suggests these individuals are otherwise indistinguishable from the other members of their communities.

Chronologically, the more mobile/nonlocals occur at Çatalhöyük in the Middle and Late periods of occupation between 6700 and 6300 cal BC (12). Only one individual likely spent more time in the paleolake basin further north or at a higher latitude than most inhabitants. Closer inspection by phase shows three females were buried during the Middle period whereas two males and two females were buried during the Late period (Fig. 3). However, although individuals may have been buried during a specific occupational phase, tooth enamel is laid down in childhood and mobile behaviors could have occurred at any point after the formation of the tooth and before burial. The date at which these behaviors change is important because the onset of the Late period of occupation at Çatalhöyük represents a fundamental shift when a number of cultural transitions also occur including changes in architecture, house decor and use of space, mobile artifacts (i.e., stamp seals), reduction in population size and density, more secondary burials, fewer burial goods, decreased aquatic resource exploitation, an increased importance of sheep and changes in husbandry, agriculture, and cooking. These changes point to an overall greater economic independence of houses (35) and a reduced interest in the commemoration of places and events (i.e., history-making) (12).

**Fig. 4.**
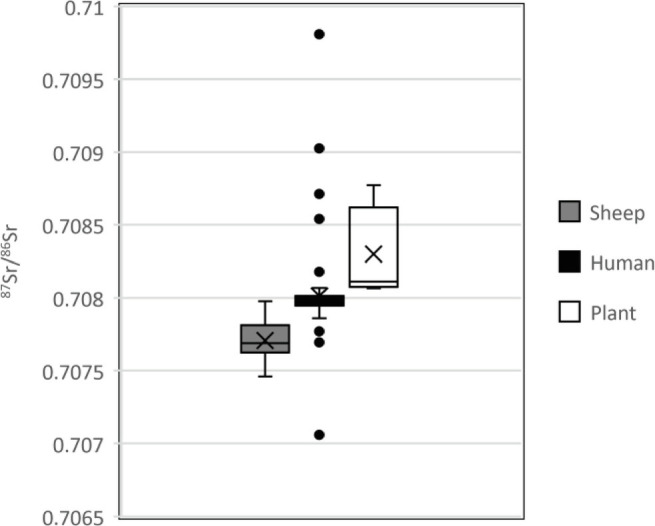
A box and whisker plot of strontium isotope values for Çatalhöyük sheep (*n* = 7), humans (*n* = 77), and plants (*n* = 7) [sheep and plant data from Bogaard et al. (39)]. British Geological Survey data © UKRI 2022.

Why these people at Çatalhöyük were mobile can perhaps be understood best by comparing strontium and oxygen isotope evidence from important foods including sheep (36) and plants (37) from the site. Although the strontium and oxygen isotope data from Çatalhöyük sheep indicate that most animals measured had values consistent with being pastured in the paleolake basin or on the terraces (38, 39), the plant strontium isotope values are consistent with gathering wild plants in more radiogenic zones. The Çatalhöyük humans largely fall within the Sr value range for both sheep and plants and this is consistent with most individuals at the site living locally within the paleolake basin as children (Fig. 4). The carbon and nitrogen isotope evidence of sheep diet from seventh millennium cal BC Pınarbaşı (a short-term hunting and herding station nearby) also suggests that the Çatalhöyük and Pınarbaşı sheep grazed in overlapping areas (38, 40).

The sheep oxygen isotope values (38, 39) highlight a further source of variation in childhood mobility, one that might indicate seasonal activities. For instance, children may have been mobile only during certain times of the year since oxygen isotope values show a 0.6‰ increase for each centigrade increase in temperature (41). However, for this to be feasible children would need to have been involved with these specific seasonal activities annually. This is supported by earlier interpretations of specialized roles for individuals at the site (42) and the increasing independence of households (12, 35), although the data presented here suggest that mobility beyond the paleolake basin was not a widespread practice but one undertaken by only a handful of individuals or groups. The single more mobile Boncuklu female is difficult to interpret in a similar fashion, but it seems likely that this individual came from or traveled further south in the paleolake basin compared to other inhabitants at the site. The lack of individuals at Pınarbaşı or Boncuklu making regular use of the limestone terraces or the Taurus Mountains does seem to indicate activities being undertaken more locally than was the case at Çatalhöyük.

Therefore, these data are consistent with largely local (within the paleolake basin) partner-exchange practices across all the sites studied over seven millennia, with some mobility, especially at Çatalhöyük, likely being the result of logistical activities in the landscape. The oxygen isotope data also point to a single possible locally mobile individual at Boncuklu. However, none were identified at Pınarbaşı at either date. These data are consistent with the interpretation that most children did not seem to accompany adults in activities that involved logistical mobility. The lack of evidence for mobility beyond the paleolake basin in the 10th to 9th millennium cal BC at either Pınarbaşı or Boncuklu is particularly surprising as it indicates that despite their small population size the avoidance of close inbreeding seen in the genetic data was avoided despite local partner-exchange practices. This is particularly compelling since several sites were contemporaneous with Boncuklu and 10th to 9th millennium cal BC Pınarbaşı both within the local area and regionally (central Turkey), but there are no contemporaneous sites locally or regionally that could have enabled exogamy at the megasite of Çatalhöyük (6, 18). These findings have important implications for how we conceptualize the early Neolithic and the extent of residential mobility that was undertaken in early, small hunter-gatherer communities more than 10,000 y ago.

## Discussion

Strontium and oxygen isotope data presented here have enabled both local and regional mobility to be determined in the development of early villages and the emergence of megasites. These data are consistent with community members occasionally moving around the landscape as children (who were the only ones to do so from their burial house), within the paleolake basin (or just beyond it) at Boncuklu and Çatalhöyük, with a small number originating from beyond the paleolake basin at Çatalhöyük. These data are also consistent with most inhabitants at these sites, certainly as children, having consumed resources grown on (or taken from) paleolake basin soils, rather than the limestone terraces. The importance of the limestone terraces as a location where crops could have been grown during wet periods at Çatalhöyük (13) is not supported by these data. The lack of more mobile individuals at the earlier sites suggests that biological kinship practices likely operated within local partner-exchange networks that also made close inbreeding unlikely. These data raise several important issues which are discussed below. First, how did early Neolithic communities balance low/local residential mobility and inbreeding risk? Second, what significance do these data have for the use of buildings/houses and sedentarizing behavior in early villages? Third, to what extent do bioarchaeological methods aid in understanding kinship behaviors?

### Low/Local Residential Mobility and Inbreeding Risk.

One of the risks of local kinship partner exchange in small early sedentary villages would have been the increased potential for close-relation inbreeding. Although the term “inbreeding” often carries negative connotations, these stem largely from modern cues as first-cousin inbreeding has been estimated to result in 1.1% excess infant deaths (43). Serious health implications occur with the greatest frequency in first-degree-related (parent–child, sibling) and second-degree-related (uncle/aunt–niece/nephew, grandparent–grandchild, double cousins) mating, depending on several factors acting on the original gene pool. More specifically, close inbreeding increases homozygosity (inheritance of identical alleles in both chromosomes), thus increasing the risk of the offspring to inherit (sometimes harmful or lethal) recessive allele mutations that under other mating practices (where individuals were less closely related) would be rarely dominant otherwise (44). It is possible that some mating behaviors in early Neolithic Turkey may have been detrimental for human populations and the resulting offspring did not survive. Although there is high infant mortality in these communities, this is likely related to high fertility rates (19). Genetic evidence from neonatal infants at Çatalhöyük and Boncuklu shows mating occurred among more distantly related individuals (33) and neonates are well represented in the burials making up a substantial proportion of the human remains recovered at both sites (45, 46).

Because the Boncuklu inhabitants were local and avoided close inbreeding, it seems that cultural or biological mechanisms existed to avoid this. Studies of contemporary egalitarian multi- or bilocal hunter-gatherers have shown how relatedness reduces where gender equality occurs in residential mobility (9, 10). Early communities may have kept track of locally mobile close relatives. It has been clear for some time that certain individuals were “tracked” after death, as attested by retrieval of specific crania/skulls in funerary practices throughout southwest Asia (47). In the Levantine PPNB the Jericho plastered skulls show cranial modification in life and plastering after death (48), showing that modified-in-life crania were retrieved for plastering and curation. The modified plastered crania also raise the issue of visual recognition of group membership since they were modified in childhood and would have been visible throughout the life course. Linked to this is the endogamous kinship at Basta that resulted in lateral incisor agenesis. Normally a rare trait, this occurred at more than 10 times the expected frequency (26). Although personal ornamentation could have differentiated between individuals, different head shapes and tooth agenesis would have been more difficult to copy and would have been highly visible cues for signaling kinship among community members.

The extent of biological relatedness (consanguinity) but avoidance of close inbreeding and incest (see refs. 49–51 for discussion of cultural versus biological debates) in Boncuklu and Çatalhöyük implies individuals were somehow classified within these communities in ways that impacted mate choice. As with chimpanzee philopatry, it might be expected that the highly mobile nature of Paleolithic communities made close inbreeding an unlikely occurrence, and yet the genetic evidence from several Neanderthals (52, 53) suggests closer inbreeding occurred and with coefficients as high as 1/8. The impact of inbreeding would have been such that groups with long-term established mating behaviors had already experienced “inbreeding depression” during the Paleolithic and deleterious alleles effectively being selected against within the population. Local mating may have continued or been reestablished during the Neolithic since we know that Çatalhöyük and Boncuklu communities are composed of largely local individuals, from a largely central Anatolian population with (chronologically) distant and limited genetic heritages from nearby regions of the Levant and the Zagros. Evidence from Ceballos et al. (54) and Ringbauer et al. (55) also suggests that population increase is also a factor here, with more distantly related groups emerging over time especially with the Neolithic Demographic Transition and the transition to agriculture.

A cultural mechanism that prevented close inbreeding must have been in place at Pınarbaşı, Boncuklu, and Çatalhöyük. This might be identified in the sociological concepts of group cohesion and maintaining the status quo (56). The avoidance of forming fixed dyads may have been part of a wider element of Neolithic society that strived to maintain social cohesion. There is extensive debate concerning the use of secondary burial practices to ensure cohesion as Neolithic communities experienced the tensions created by changes that cut across material culture, population size, and food acquisition. At Çatalhöyük we observe an increase in these practices in the Late period, one that was associated with key sociopolitical transitions described above. Based on reanalysis of contemporary Israeli kibbutzim data (including contemporary interviews) Shor and Simchai (56) argue that the stronger the associations between individuals within delimited groups the more likely they were to be sexually indifferent to one another. Therefore, recognizing and tracking historical relationships between individuals and the nature of social relationships between groups may have been part of the process that prevented individuals from mating with close biological kin.

### The Use of Houses and Sedentarizing Behavior in Early Villages.

Little is known about the dynamic way that houses were used by both the living and the dead in terms of how long specific individuals inhabited or used a particular house, how often they moved between them, and if houses used by the living influenced the choice of house for subfloor burial of the dead. At both Çatalhöyük and Boncuklu floors were repaired after a burial occurred and use of the house continued (6, 12). House choice for burial might also represent long-term affiliation of an individual with that house or a more immediate, short- or medium-term arrangement (57). Ethnographic evidence shows that among contemporary hunter-gatherer populations, including Indigenous Australians, the Hadza of Tanzania, and the Ju/’hoansi of the Kalahari, vary their residence according to age. Younger couples often live with the wife’s family, whereas older couples often rejoin the husband’s residence as families grew (58). This is an important point because isotopic evidence of diet at Çatalhöyük has shown that community-wide food sharing was predicated on age, with younger adults (<*ca*. 30 y of age) consuming a different diet than older individuals (59). Provisioning different groups with food according to age, in sufficient quantity to impact the carbon and nitrogen isotope values of adult bone collagen, indicates there was a major commitment to differentiating between them regularly, perhaps daily, and would have been more feasible through household-based, rather than community-wide, meals.

A further issue concerns the present understanding of the nature of prehistoric male–female partnerships within kinship practices, which are likely oversimplified. These have been implicitly assumed to be single and monogamous, whereas serial monogamy or polyandry/polygyny-style practices have not been discussed. The extent to which these were practiced in prehistory has not been demonstrated in prehistoric southwest Asia. This poses an obstacle to understanding the function of houses and households from buried individuals since some individuals may have had strong affiliations to several households or none. At Çatalhöyük although almost all houses contained burials, some houses had very few, the highest number occurred in Building 1, which contained 60 individuals (60). At Boncuklu house burials range in number from none to six. Similarly, some houses at Çatalhöyük are associated with extensive feasting deposits but are otherwise indistinguishable from others. At Boncuklu primary inhumations, secondary burials, and structured deposits of scattered human remains unassociated with houses indicate that some individuals were not afforded a within-house burial as their final interment (6, 46). At Boncuklu, coburial in a sequence of houses of first-degree-related adults does occur, albeit in a limited sample (33). While related adults seem to be associated with particular houses, the evidence suggests there is no simple correlation between nuclear family occupation and burial in houses. In contrast, at Çatalhöyük genetic evidence for parents and children or siblings buried in the same house is uncommon, although there is limited ancient DNA evidence for adults being related. This evidence seems to suggest that, particularly for Çatalhöyük and other Late Neolithic sites (e.g., Barcın), the use of houses for nuclear or even extended families as suggested by Flannery (20) and Kuijt et al. (61) was unlikely to have been that straightforward and stood in contrast to earlier Neolithic practices. The decrease in the degree of genetic relatedness between those coburied in houses at Boncuklu and Çatalhöyük is paralleled in the potential increased appearance at Çatalhöyük of at least modest numbers of individuals from outside the immediate area around the megasite. It seems likely that increasing flexibility and variability in kinship arrangements and coresidence contributed to megasite community growth and household organization.

### Using Bioarchaeological Methods to Understand the Range of Kinship Behaviors.

Most bioarchaeological research has focused on finding positive evidence for biological relatedness among individuals, especially among those buried in houses. However, this is only part of the story (62) and only recently has the concept of the importance of biological kin versus “fictive” kin (a form of social kinship that involves relationships based on neither biological nor affinal, i.e., marriage-like, ties) been suggested for prehistoric southwest Asia (e.g., refs. 24 and 63). If individuals that are buried together show little or no biological relatedness, then social or political reasons for their coburial were likely to have been key factors in the decision-making behind burial location. If fictive kin are indeed present in house burial throughout the Neolithic, how does this inform understandings of the use of houses by these communities?

The anthropological concept of house-based society at this point serves as a useful heuristic device for discussion since such communities are not organized primarily into lineal descent groups and instead existed in a variety of forms. Neolithic societies also potentially represent examples of social practices that no longer exist in their entirety since contemporary and ethnographic examples are many thousands of years removed (57). Yet, several features of communities organized around houses highlight complexities in social organization that may contribute toward an understanding of the association between buried individuals and houses in the Neolithic. Houses in Neolithic southwest Asia were long-term structures that outlived the individuals buried there and likely formed part of a complex suite of behaviors that tied individuals together sociopolitically. Even with building renewal, at both Çatalhöyük and Boncuklu, diversity in the genetic heritage of the individuals buried beneath these structures was maintained. Kinship behaviors may have been the result of membership recruitment through social or political means rather than the other way around (57). This would result in multiple systems of partner exchange, coresidence patterns, and burial practices and would help to explain the diversity of practices seen in Çatalhöyük, Boncuklu, and the wider Neolithic, especially during the PPNB in the Levant. Burial practices could be seen as a means to maintain sociopolitical connections with those who came before, with identities reflected in death as they were in life as seen in plastered skulls across southwest Asia (48) and choice of burial location reflecting distinctions in diet during life as seen elsewhere in Neolithic Turkey (63). The desire to maintain social cohesion with fictive kin through fluid house associations that fluctuated throughout the life of the structure, but not following prescribed rules of relatedness according to particular circumstances, brings us a step closer to understanding the various and diverse ways that communities were organized around houses in the world’s first villages.

A striking element of this study, and other bioarchaeological kinship studies, is the diversity of practices employed both within and between each site studied. This variation highlights a critical issue in kinship studies of Neolithic southwest Asia, namely that sites (including megasites) arose employing complex, often unique and varied, practices to build their own communities. It also highlights an issue with archaeological research into kinship practices more widely: that attempts to pigeonhole these early communities miss an important element of their partner-exchange practices that did not prioritize or seek to reinforce concepts of biological relatedness but rather emphasized shared ideologies and associations involving fictive kin as a key component of social organization.

Increasing sedentism in southwest Asia 10,000 y ago had a profound impact on human lifeways, gradually altering economic and social behaviors that ultimately reduced mobility. At this point in the early Holocene villages were small. Yet, within 1,000 y megasites occupied by thousands of inhabitants appeared. Bioarchaeological evidence had previously indicated population growth, and the strontium and oxygen isotope data presented here suggest communities were built largely in situ, with mobility principally tied to foraging, cultivation, and herding practices. Yet, genetic data indicate a lack of close inbreeding and only limited biological relationships among individuals buried in the same house. These findings suggests that small sedentarizing villages of the early Neolithic had highly localized kinship networks that negated the need for strict matrilocal or patrilocal residence practices. Shifting the focus to the use of houses, the variety of relationships and the lack of clearly prescribed kinship practices suggests that megasites may have arisen by employing unique, community-specific kinship practices that were based on sociopolitical relationships that likely owe more to fictive rather than biological kinship.

## Materials and Methods

Following established principles and methods (64–66), we used a combination of new and recently published ancient and modern data to characterize the paleolake basin, a geologically homogeneous alluvial former lake basin where the sites are located (see *SI Appendix*, *SI Text 5* for full details of the materials and methods). We chose M2 and M3 teeth and sampled the following percentages of the available population: Pınarbaşı Epipaleolithic 1/3 (33%); for Pınarbaşı 10th to 9th millennium cal BC 3/6 (50%); for Boncuklu 18/40 (45%); and for Çatalhöyük 77/741 (10%). These represent a demographic profile of 23 adolescents, 26 young adults (20 to 30 y), 26 middle adults (30 to 49 y), 12 old adults (50+ y), and 12 adults for whom age could not be estimated precisely. Samples were prepared at the National Environmental Isotope Facility, British Geological Survey, Keyworth, UK. Oxygen isotope values are reported as δ^18^O in per mil. (‰) (^18^O/^16^O) normalized to the VPDB scale using a within-run calcite laboratory standard (KCM) calibrated against NBS-19 and NBS-18 IAEA reference materials. Analytical reproducibility for δ^18^O_VSMOW_ is ±0.06‰ (1σ,) and δ^13^C_VPDB_ ± 0.03‰ (1σ). External reproducibility of the enamel data, based on the analysis of 18 duplicate sample pairs, is ±0.1‰. Strontium isotope values are reported relative to the international standard for ^87^Sr/^86^Sr, NBS987, which gave a value of 0.710258 ± 0.000020 (two SD, *n* = 8) during the analysis of these samples. This is within uncertainty of the accepted value of 0.710250 and hence the data are uncorrected relative to the standard.

## Supplementary Material

Appendix 01 (PDF)

## Data Availability

All study data are included in the article and/or *SI Appendix*.
