# Supplementary material for: Mobility and kinship in the world’s first village societies
Source: Proc Natl Acad Sci U S A. 2023 Jan 17;120(4):e2209480119. doi: 10.1073/pnas.2209480119 (PMC9942817; doi:10.1073/pnas.2209480119)
Supplement: Supplementary file 1 — Appendix 01 (PDF) [file pnas.2209480119.sapp.pdf]

PNAS

Supplementary Information for:

Mobility and Kinship in the World's First Village Societies

Jessica Pearson[a*]
Jane Evans[b]
Angela Lamb[b]
Douglas Baird[a]
Ian Hodder[c]
Arkadiusz Marciniak[d]
Clark Spencer Larsen[e]
Christopher J. Knüsel[f]
Scott D. Haddow[g]
Marin A. Pilloud[h]
Amy Bogaard[i,j]
Andrew Fairbairn[k]
Jo-Hannah Plug[a]
Camilla Mazzucato[g]
Gökhan Mustafaoğlu[l]
Michal Feldman[m, n]
Mehmet Somel[o]
Eva Fernández-Domínguez[p]

[*] Corresponding author: Jessica Pearson, Department of Archaeology, Classics and Egyptology, 12-14 Abercromby Square, University of Liverpool, Liverpool, L69 7WZ, UK. pearson@liv.ac.uk

[a]Department of Archaeology, Classics and Egyptology, University of Liverpool, Liverpool, L69 7WZ, UK
[b]National Environmental Isotope Facility, British Geological Survey, Keyworth, Nottingham, NG12 5GG, UK
[c]Archaeology Center, Department of Anthropology, Stanford University, Palo Alto, CA 94305
[d]Faculty of Archaeology, Adam Mickiewicz University, 61-614 Poznań, Poland
[e]Department of Anthropology, The Ohio State University, Columbus, OH 43210
[f]UMR-5199 De la Préhistorie á L'Actuel: Culture, Environnement, et Anthropologie (PACEA), University of Bordeaux, 33615 Pessac Cedex, France
[g]Department of Cross-Cultural and Regional Studies, University of Copenhagen, 2300 Copenhagen S, Denmark
[h]Department of Anthropology, University of Nevada, Reno; Reno, NV 89557
[i]Institute of Archaeology, 36 Beaumont Street, University of Oxford, Oxford, OX1 2PG, UK
[j]Santa Fe Institute, Santa Fe, NM 87501
[k]School of Social Science, The University of Queensland, Michie Building, St Lucia, Brisbane, QLD 4072, Australia
[l]Department of Archaeology, Faculty of Letters, Ankara Hacı Bayram Veli University, Abant 1 Cad. No:10/2D, Yenimahalle, 06570, Ankara, Turkey
[m] Archaeo- and Palaeogenetics group, Institute for Archaeological Sciences, University of Tübingen, 72074 Tübingen, Germany

[n] Senckenberg Centre for Human Evolution and Palaeoenvironment, University of Tübingen, 72074 Tübingen, Germany
[o] Department of Biological Sciences: Biology/Molecular Biology and Genetics, METU, 06800 Ankara, Turkey
[p] Department of Archaeology, University of Durham, Durham, DH1 3LE, UK

Email: pearson@liv.ac.uk

This PDF file includes:

Supplementary text: SI Text 1 to SI Text 5
Figure: S1 to S2
Tables: S1 to S3
SI References

SI Text 1: Archaeological Summaries of Çatalhöyük, Boncuklu and Pınarbaşı

Archaeological background of Epipaleolithic Pınarbaşı
The multi-period site of Pınarbaşı, comprising a series of rock-shelters and caves and an open-air tell site is located approximately 30km southeast of Boncuklu and 25 km southeast of Çatalhöyük on the edge of the Konya Basin by the Bozdağ limestone hills, northwest of the Karadağ mountain. This site has the only excavated Epipaleolithic deposits in central Anatolia. Early excavations in the mid 1990s were led by Professor Trevor Watkins (Edinburgh University) while the remains analyzed here form part of the more recent excavations in the 2000s led by Professor Douglas Baird (Liverpool University), which led to the discovery of the Epipaleolithic deposits. The Epipaleolithic deposits are located in one of the rock shelters, while the later 10th-9th millennium cal BC deposits are located in an open-air site identified as a small mound just tens of meters away from the rock shelter. The community at Epipaleolithic Pınarbaşı have been previously described as “probably highly mobile and low density” (1) and likely moved around the central Anatolian Plateau and as far south as the Taurus mountains for resources (2). Tasks involving mobility would have included food acquisition. The archeozoological evidence indicates the inhabitants of this site hunted wild sheep, goats and cattle, and caught fish and wetland birds. In terms of food plants, there is no evidence for hackberry, almond, cultivated cereals or their wild ancestors and thus plant consumption would have focused on other wild gathered resources although these were rare. Only one terebinth nutshell fragment and one *Lens* sp. seed document likely plant species collected for food. Groundstone tools were not common and consisted of grooved stones and other tool manufacturing items unlikely to have been used in plant processing. There was only one quern/grinding stone fragment that might have been connected to plant processing although other uses are also possible (2). In terms of burials, three individuals have been recovered including two *in situ* adult males and fragments of a third individual. Of these only one provided a suitable tooth (adult M2 or M3). Direct radiocarbon dating of one individual, who also yielded genomic information, resulted in a date of 13,646-13,284 cal BC (2 sigma range 95.4%) (1). Overall radiocarbon dates indicate an Epipaleolithic site occupied between 14,000 and 11,000 cal BC (2) likely pre-dating and contemporaneous with the earliest phases of the Levantine Natufian.

Archaeological background of 10th-9th millennium cal BC Pınarbaşı
The 10th-9th millennium cal BC open air site of Pınarbaşı is just tens of meters away from the Epipaleolithic rock shelter and measures approximately 1 ha in extent. Buildings were suboval and constructed of wattle and daub. Although the presence of buildings suggests a more sedentary population relative to the Epipaleolithic, the plant and animal remains of the community show no evidence for cereal and legume consumption or cultivation nor the presence of domestic animals as seen at later Neolithic Çatalhöyük (3). Wild cattle as with Boncuklu were important, but with a crucial difference that sheep and goats were too, plus equids and wild boar. Wetland

resources were present but in much reduced importance relative to Boncuklu. Radiocarbon dating indicates the earliest deposits date to 9800-9400 cal BC with occupation throughout much of the ninth millennium cal BC. The latest occupied horizons date to between 8200-7800 cal BC. This is broadly contemporary with the Levantine PPNA through to the middle PPNB (3). In terms of burials only six have been recovered, comprising both adults and subadults and of these three provided a suitable sample tooth (adult M2 or M3). The site predates early Neolithic Boncuklu by several centuries, but these two sites were likely contemporary for 300-500 years approximately (3). That the two sites could have been used by the same population has been ruled out from studies of the seasonality of plant and animal remains, especially the exploitation of birds at both sites, which confirms continuous occupation throughout much of the year and attests to limited mobility of the community too. Further evidence of two distinct communities is provided in terms of personal ornaments (4) architecture and ritual behavior (5).

Archaeological background of Neolithic Boncuklu
The early Neolithic site of Boncuklu is located on the Konya Basin approximately 30km northwest of Pınarbaşı and 10km northeast of Çatalhöyük. Ongoing excavations directed by Professor Douglas Baird (Liverpool University) began in 2006. Like the 10th-9th millennium cal BC at Pınarbaşı the site spans approximately 1 ha, but buildings were more substantial than with mudbrick construction but still in the form of a suboval shape rather than the rectilinear buildings seen at later Çatalhöyük. The earliest radiocarbon dated horizons indicate occupation at least from 8300-8100 cal BC with the latest dated phases so far pointing to occupation up to around 8100-7800 cal BC (3). Direct dating of human remains also confirms occupation in the ninth millennium cal BC. Much of the diet would have been provided by wild cattle, boar, fish and wetland birds as well as plant foods predominantly from nuts and fruit. Geomorphological data indicate food resources were likely exploited within the local environment from the wetland steppe environment present in the early Holocene, which corresponds broadly with the archaeology of the early Neolithic. There is also some evidence for small-scale cultivation of cereals and legumes, which have been directly radiocarbon dated and confirmed to date to the ninth millennium cal BC (3). DNA evidence seems also to confirm the lithic evidence at the site that the population is represented by an *in situ* local hunter-gatherer community (3) that adopted farming. In terms of burials there have been at least 40 burials to date, comprising both adults and subadults of which 18 provided suitable tooth samples (adult M2 or M3).

Archaeological background of Neolithic Çatalhöyük
The later Neolithic site of Çatalhöyük is also located in the Konya Basin of Central Anatolia approximately 10km southwest of Boncuklu and 25km northwest of Pınarbaşı. It was discovered in the 1950s and originally excavated by James Mellaart in the 1960s. Renewed excavations took place between 1993-2017 under the direction of Professor Ian Hodder (Stanford University). The site is comprised of substantial agglomerated rectilinear mudbrick houses, which is a contrasting building shape and technique compared to the earlier sites. The site covers approximately 13 ha, which is also significantly larger than all other sites in Anatolia and most other sites in southwest Asia at this time. Extensive radiocarbon dating has identified the earliest occupation dating from 7100 through to 5950 cal BC (6). The site is particularly well known for an extensive range of ‘mother goddess’ figurines, which are now thought to signal a preoccupation with the ageing body (7). Wall paintings, wall reliefs and use of cattle bucrania were used to decorate houses. Domestic cattle and sheep were the most important animal resources in the diet, followed by goats, and wild resources including aurochs, wild boar, equids and very small numbers of deer (8). The exploitation of plant remains included a mosaic of domesticated, wild and those in the process of domestication resources (9). In terms of burials, more than 700 individuals have been recovered comprising the full range of ages-at-death, but a substantive number of these did not yield a suitable tooth (adult M2 or M3) for analysis. In total 77 samples were collected.

SI Text 2: The bioarchaeological context of Neolithic southwest Asian kinship practices
Large-scale paleodemographic studies and region-specific studies of the Levant (10, 11) demonstrate that site size and population density increased exponentially during the 8th millennium cal BC (middle and late PPNB). At some mega-sites, such as Çatalhöyük, population

increase can be observed *in situ* (12). Significant changes to architecture also occurred, including the appearance of larger and two-storey structures and increased sub-division of houses (11, 13). Kuijt (11) attributes the added compartmentalization of houses to scalar stress from the impact of population increase on the wellbeing of community members and the need for privacy as well as for storage of increasing amounts of foodstuffs and material goods. The most common funerary practice in Neolithic southwest Asia was to bury the dead beneath or close to a house (14). Because these houses were initially small, incorporating a hearth and evidence of food preparation, and a sleeping area large enough for only a handful of individuals, it has been tempting to assume early communities were comprised of nuclear families each living in a house they had constructed and under which they were buried when they died. For example, Flannery (15) argued the first communities began as corporate groups prior to the PPNB, and that nuclear families emerged during the PPNB and only developed into extended nuclear families towards the end of the Neolithic as much larger houses emerged and children remained associated with their natal houses. Banning and Byrd (13) suggested nuclear or small-scale households occurred throughout the Levantine PPNA and PPNB, with changing house-size reflecting the emergence of economically autonomous households in the PPNB. More recent analyses have explored more complex arrangements including house societies (16) and have questioned the idea of autonomous households (5).

Architectural and site survey evidence offer limited opportunities to investigate the relationships between specific individuals. Bioarchaeological methods now offer insight into genetic relatedness and residential behaviors that allow us to assess past kinship behaviors determined directly from human remains. Through a combination of genetic, stable isotope, and morphological trait analysis it is possible to address relatedness between individuals and within individual houses in order to establish biological affinity and the type of kinship practices adopted. Early research in southwest Asia focused largely on non-metric dental traits at sites such as Abu Hureyra (17) and Çatalhöyük (18) where family burial was argued for. In contrast, Pilloud and Larsen's (19) study of the newly excavated Çatalhöyük skeletal assemblage using dental metric and morphological analysis did not show evidence for biological affinity correlated with burial co-location either within houses or in neighborhood clusters, but instead represented "practical kin" (non-genetic or affinal relations) associations. Alt et al. (20) observed dental traits in individuals from PPNB Kfar HaHoresh and tentatively concluded that spatial correlation of these traits indicated family plots, a closer genetic relationship between adolescent and adult females during the late period of occupation, and greater trait diversity in females. However, the authors were unable to confirm these indicators were evidence for matrilocality. At the PPNB mega-site of Basta, Alt et al. (21) used a similar approach combined with strontium isotope analysis to reveal inherited trait frequency that strongly suggested local, within-community inbreeding and deliberate endogamous kinship practices. Santana et al. (22), also measured strontium isotopes alongside those of oxygen and carbon isotopes in sites spanning the Natufian to the PPNC in the Southern Levant and suggested greater mobility prior to the Neolithic but with the mega-site of 'Ain Ghazal showing individuals were local to the area. The practices at Basta and 'Ain Ghazal provide a valuable counterpoint to the evidence from Çatalhöyük in understanding the kinship mechanisms that gave rise to mega-sites and, ultimately, the origins of urban living.

In southwest Asia regional studies of ancient DNA have enabled examination of the earliest inhabitants of the region to determine genetic relationships with and between sites. Lazaridis et al. (23) and Kılınç et al. (24) documented regional genetic continuity between local hunter-gatherers and farmers in both the Levant and the Zagros, where populations were more closely related to earlier local groups than populations at more distant but contemporary sites. These findings suggest that archaeological evidence for long-distance exchange networks trading resources such as Central Anatolian obsidian (25) and Mediterranean shells (2), may not have involved large-scale movements of people for partner-exchange. Although this appears to run counter to the findings of Santana et al. (22) who argued that Natufian populations likely originated from the same region as imported obsidian.

The earliest genetic evidence from Anatolia dates to the late Pleistocene. Feldman et al. (1) demonstrated that the Epipaleolithic population from Pınarbaşı shows evidence for earlier genetic connections between Levantine, Anatolian and European hunter-gatherer populations. At early Neolithic Boncuklu the genetic data show that around 90% of the population's genetic heritage was derived from a population similar to that of Epipaleolithic Pınarbaşı. These findings suggest an indigenous development of the Neolithic populations that lasted for at least seven millennia. The origin of the population that contributed to the remaining 10% of the genetic heritage at Boncuklu may have derived from a population that inhabited the region of modern-day Iran in the late Pleistocene or early Holocene C. 11,500 -8,500 cal BC (1). Moreover, this study shows that Neolithic Levantine populations, represented by individuals from Kfar HaHoresh, Ba'ja and 'Ain Ghazal, received gene flow from Anatolian populations during the early Holocene, comprising around 20% of their genetic heritage. This was followed by Levantine gene flow of a similar extent entering western Anatolia during the Late Neolithic. These data support small scale but regular mobility in southwest Asia throughout the Neolithic.

At Çatalhöyük Chyleński et al. (26) revealed that the individuals buried there showed closer genetic relationships with other Anatolian populations, especially the Marmara Region (NW Turkey), compared to contemporaneous Levantine populations. This finding suggests that the Çatalhöyük population likely originated from indigenous Central Anatolian hunter-gatherers and early farmers, at least in the later levels. Within the site itself, Chyleński et al. (26) recovered 10 mitochondrial genomes and several different haplotypes. Four individuals, each with different haplogroups, were buried beneath a single house indicating the presence of at least four different maternal lineages and no evidence of biological relatedness among them, suggesting either a large patrilocal or non-genetic kin-based society. Most recently, Yaka et al. (27) used genetic data from 59 individuals to test the hypothesis that Neolithic individuals buried beneath the same house were closely-related genetically, but found limited evidence for this among co-buried individuals at both Çatalhöyük (n=14) and Barcın Höyük (n=23). However, they argue there may have been a reduction in co-burial of genetic relations through time since higher prevalence of co-buried first-degree relatives was found in the earlier Neolithic at Boncuklu (n=9) and Aşıklı Höyük (n=8). Both earlier communities practiced within house co-burial of siblings and parents with children. This study also provides an inbreeding coefficient for three of the sites, which indicated 1/32 relatives (i.e., first cousins once removed) or less in the earlier Neolithic (Aşıklı Höyük and Boncuklu) with a lower co-efficient (more distantly related still) in the later Neolithic at Barcın Höyük (<1/64= i.e., second cousins).Ringbauer et al. (28) published a comparative global study of runs of homozygosity (ROH), which indicate the degree of parental relatedness, from genomic data of 1785 individuals buried over 45,000 years (including reporting data from Epipaleolithic Pınarbaşı and Boncuklu) in spatio-temporal context. These data showed that ROH have reduced over time and there was a generally low rate of first cousin or closer inbreeding, which would result in ROH values >50cM (centimorgan). Both Epipaleolithic Pınarbaşı and Boncuklu show ROH was <20cM as did data for other Neolithic sites in southwest Asia in the study. Unfortunately, the sequenced data for our third study site Çatalhöyük has no published evidence for ROH at this time but remains an important avenue for future research.

SI Text 3: Geological summary

Pınarbaşı is located at the interface between the limestone terraces and the Pleistocene marl substrate that characterizes the eastern edge of the Konya basin, at the edge of the Karadag volcanic massif and is c. 30km southeast of Boncuklu. Boncuklu is located in the middle of the Konya basin and was founded directly on the lake marl, with pockets of water-laid sediment accumulating around it during its occupation. Çatalhöyük is also located in the center of the Konya basin 10km south of Boncuklu and 25km northwest of Pınarbaşı and was founded during the active formation of an alluvial fan over the marl substrate. See Figure 1 in the main text for a location map of the sites and local geology.

SI Text 4: Extended discussion of strontium and oxygen isotope results

Bogaard et al. (29) reported paleolake basin alluvial soil $^{87}Sr/^{86}Sr$ ratios between 0.7076 and 0.7081 with a gradual cline between Çatalhöyük and Boncuklu and lower values occurring

towards the south of the paleolake basin. We used these Sr values as a cut-off to distinguish between local individual values, which would be consistent with the paleolake basin values and non-local individuals, which would fall outside of this range. The plants measured from the limestone terraces close to Pınarbaşı overlap slightly with the paleolake basin values and range from 0.7069 to 0.7076. Soil leachates from Pınarbaşı burial sediments (measured as part of this study) fall within the range for the basin soils with an average value of 0.7079 from two virtually identical measurements (0.70787 and 0.70788). The results from Boncuklu and Pınarbaşı are shown in the main text in Figure 2 and Çatalhöyük in Figure 3. A total of 92 out of 99 individuals fall within the paleolake basin soils range and thus are consistent with an early childhood spent in this area. Several non-local individuals (those with values beyond the paleolake basin soil range) were found at Çatalhöyük, while at Boncuklu and Epipaleolithic and 10th-9th millennium cal BC Pınarbaşı these data were consistent with all individuals deriving from the paleolake basin.

The non-local individuals at Çatalhöyük have $^{87}Sr/^{86}Sr$ ratios both higher and lower than those for the paleolake basin. This is consistent with a childhood spent either in, or occasionally travelling to, the Taurus Mountains (higher radiogenic zone) or the limestone terrace (lower radiogenic zone). However, because much of the paleolake basin alluvium derives from the Taurus Mountain outcrops (29), the individuals with only slightly higher $^{87}Sr/^{86}Sr$ ratios who fall outside of the paleolake basin range are also consistent with regular short-term mobility between the paleolake basin and Taurus Mountains, or they originate from a settlement between these two geologic zones. In addition, the data from one individual is consistent with them having spent part of their childhood in the limestone terraces. At Pınarbaşı, which is closest to the limestone terraces, the data from all four individuals (three from the 10th- 9th millennium cal BC and one from the Epipaleolithic periods of occupation) are consistent with childhoods spent in the paleolake basin (rather than the terraces).

Using oxygen isotope values from the Global Network of Isotopes in Precipitation (GNIP) the predicted mean $\delta^{18}O_{(VSMOW)}$ values for precipitation in Turkey are -7‰ to -9‰ (30), with a weighted annual mean of -8.4‰ and a seasonal range between a minimum of -12‰ (winter) and a maximum of -4‰ (summer) (31). To calculate the expected human values, we used published equations (See SI Text 5) to provide comparable data to characterize the region. The mean $\delta^{18}O$ values for both Boncuklu ($\delta^{18}O_c$ 25.79‰, $\delta^{18}O_p$ 16.94‰) and Çatalhöyük ($\delta^{18}O_c$ 25.88‰, $\delta^{18}O_p$ 17.03‰) individuals are virtually identical (i.e. within machine-error) to each other and fall within the annual winter-summer range for central Turkey regardless of the equation used. These data are consistent with inhabitants at both sites spending their childhood in the same approximate area (excluding the non-local/more mobile Sr isotope individuals). Using the oxygen isotope value IQR (Table S1) to detect individuals who spent time in, or originated from, different parts of the landscape; only one or two seem to occur at each site. This is despite the fact the range is more constrained at Çatalhöyük, although this could also be due to a larger sample size.

At Boncuklu there is no difference in the mean values of males and females, although one female among the oxygen isotope data is consistent with a childhood spent further south in the paleolake basin. Significantly, there is one individual that falls within the IQR for the other Boncuklu inhabitants, but these data are also consistent with them having spent their childhood in an area that overlaps with the inhabitants from the Pınarbaşı 10th-9th millennium occupation, which are consistent with a childhood spent in a more northerly region of the paleolake basin. Since Pınarbaşı is in a more southerly region of the paleolake basin on the edge of the terraces, this might indicate seasonal wholesale movement by the inhabitants. A specific area of the paleolake basin may have been used by inhabitants from both settlements, or the Boncuklu individual might have originated from the Pınarbaşı community or from another community using the same environmental zones. In contrast, the data for the Pınarbaşı Epipaleolithic individual is consistent with a childhood spent in the paleolake basin but likely further south than did the occupants of Pınarbaşı during the 10th-9th millennium. At Çatalhöyük the individuals with Sr values data consistent with greater mobility or being non-local rarely coincide with more mobile oxygen isotope values, suggesting those with strontium isotope values consistent with mobility moved across zones at a similar latitude and/or altitude as the paleolake basin.

SI Text 5: Materials and Methods

Principles of strontium isotope analysis

Strontium (Sr) occurs naturally in soils and enters the food chain predominately through plant uptake. Humans ingest strontium primarily through plant consumption, but also from water and other foodstuffs. Strontium is deposited in tooth enamel, substituting for calcium during the formation of teeth in childhood. $^{87}Sr/^{86}Sr$ compositions vary geographically according to differences in the age and chemistry of the underlying rocks that supply the inorganic components of soils (32, 33, 34). To identify non-local individuals, we used a combination of new local soil values reported here and recently published ancient plants and modern soil values to characterize the paleolake basin (29), a geologically homogeneous alluvial former lake basin where these sites are located.

Principles of oxygen isotope analysis

The oxygen isotope composition of rainwater varies systematically across the globe. Thus, drinking water that is unmodified by mixing or further fractionation reflects the local averages for specific areas, and the values measured in tooth enamel can be related to local drinking water values and used as a geographic marker (35). The oxygen isotope composition of tooth enamel is compared directly against established datasets for particular areas (30). Culturally-mediated modification can change the value of ingested water through boiling and stewing (36), and post-weaning milk consumption can also affect values (37). In assessing oxygen isotope data, potential water sources and these moderating effects must be considered.

Sampling

Most samples were taken from permanent second molar teeth (enamel formation: 2.5-8.5 years) or occasionally from a third molar tooth (enamel formation: 8.5-14.5 years) (38) when the antimere was present. Half the crown was removed (mesio-distally) for analysis. This approach enabled future morphological or isotopic work to be undertaken on the remaining tooth, or its antimere, thus helping to preserve future research opportunities. To ensure comparable data, we chose teeth that exhibited little wear and no carious lesions, and no obvious signs of developmentally-related enamel defects. This sampling strategy provided samples from the following: 77 individuals for Çatalhöyük, 18 for Boncuklu, three for Pınarbaşı 9th millennium cal BC occupation, and a single individual for Epipaleolithic Pınarbaşı. The enamel surface of the tooth was abraded to a depth of >100 microns using a tungsten carbide dental bur and the removed material discarded. An enamel sample was cut from the cleaned area of the tooth using a flexible diamond edged rotary dental saw. All surfaces were mechanically cleaned with a diamond coated bur to remove adhering dentine.

Chemical preparation and isotope analysis of oxygen in enamel structural carbonate

Approximately 3mg of enamel was reduced to powder in an agate mortar and pestle. The powder was subsequently loaded into a glass vial and sealed with septa. The vials were transferred to a hot block at 90°C on a GV Multiprep system. The vials were evacuated, and 4 drops of anhydrous phosphoric acid were added. The resultant CO_2 was collected cryogenically for 15 minutes and transferred to a GV IsoPrime dual inlet mass spectrometer at the National Environmental Isotope Facility, British Geological Survey, Keyworth, UK. The resultant isotope values were treated as a carbonate. Isotope values are reported as $\delta^{18}O$ ($^{18}O/^{16}O$) in per mille (‰) normalized to the VPDB scale using a within-run calcite laboratory standard (KCM) calibrated against NBS-19 and NBS-18 IAEA reference materials and converted to the VSMOW scale using the published conversion equation (39): VSMOW= (1.03091 x $\delta^{18}O_{VPDB}$) +30.91. To calculate the expected human values, we used the equations in SI Table S2 to provide comparable data to characterize the region. Next, we converted the human $\delta^{18}O_{carbonate}$ ($\delta^{18}O_c$) values to $\delta^{18}O_{phosphate}$ ($\delta^{18}O_p$) values using the following equation reported in Chenery et al. (40):

$$\delta^{18}O_{phosphate}=1.0322 \times \delta^{18}O_{carbonate} - 9.6849$$

Then we used the interquartile range (IQR), following Lightfoot and O'Connell (35), to provide conservative end point estimates for identifying evidence for latitudinal mobility within the paleolake basin. Altitudinal effects were excluded from interpretations within the basin due to the flat nature of this region having been formed from a dried-out Pleistocene paleolake not subsequently altered by folding or faulting (41).

Chemical preparation and isotope analysis of strontium in enamel:
Samples were transferred to a clean (class 100, laminar flow) working area for further preparation. In a clean laboratory, the sample was first cleaned ultrasonically in high purity water to remove dust, rinsed twice, and then soaked for an hour at 60° C, rinsed twice, then dried and weighed into pre-cleaned Teflon beakers. The sample was mixed with ^{84}Sr tracer solution and dissolved in Teflon distilled 8M HNO_3 and converted to chloride form using 6M HCl. Strontium was collected using Eichrom AG50 X8 resin columns. Strontium was loaded onto a single Re Filament following the method of Birck (42) and the isotope composition and strontium concentrations were determined by Thermal Ionization Mass spectroscopy (TIMS) using a Thermo Triton multi-collector mass spectrometer at the National Environmental Isotope Facility, British Geological Survey, Keyworth, UK.

SI Figure S1: Locations of individuals measured for strontium and oxygen isotope analysis from the North shelter excavations at Çatalhöyük. Circles indicate local individuals. Diamonds indicate more mobile individuals.

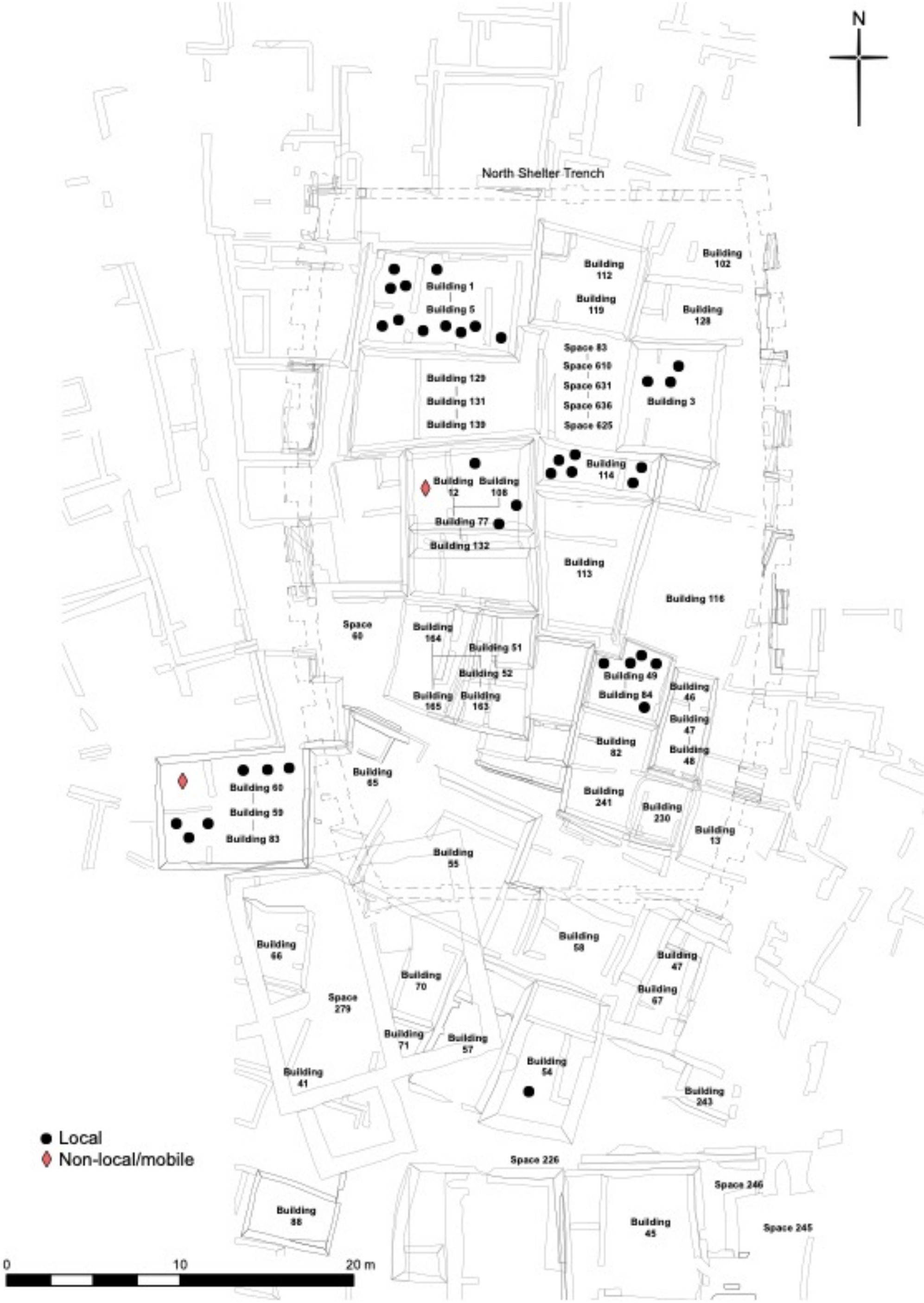

SI Figure S2. Locations of individuals measured for strontium and oxygen isotope analysis from the South shelter excavations at Çatalhöyük. Circles indicate local individuals. Diamonds indicate more mobile individuals.

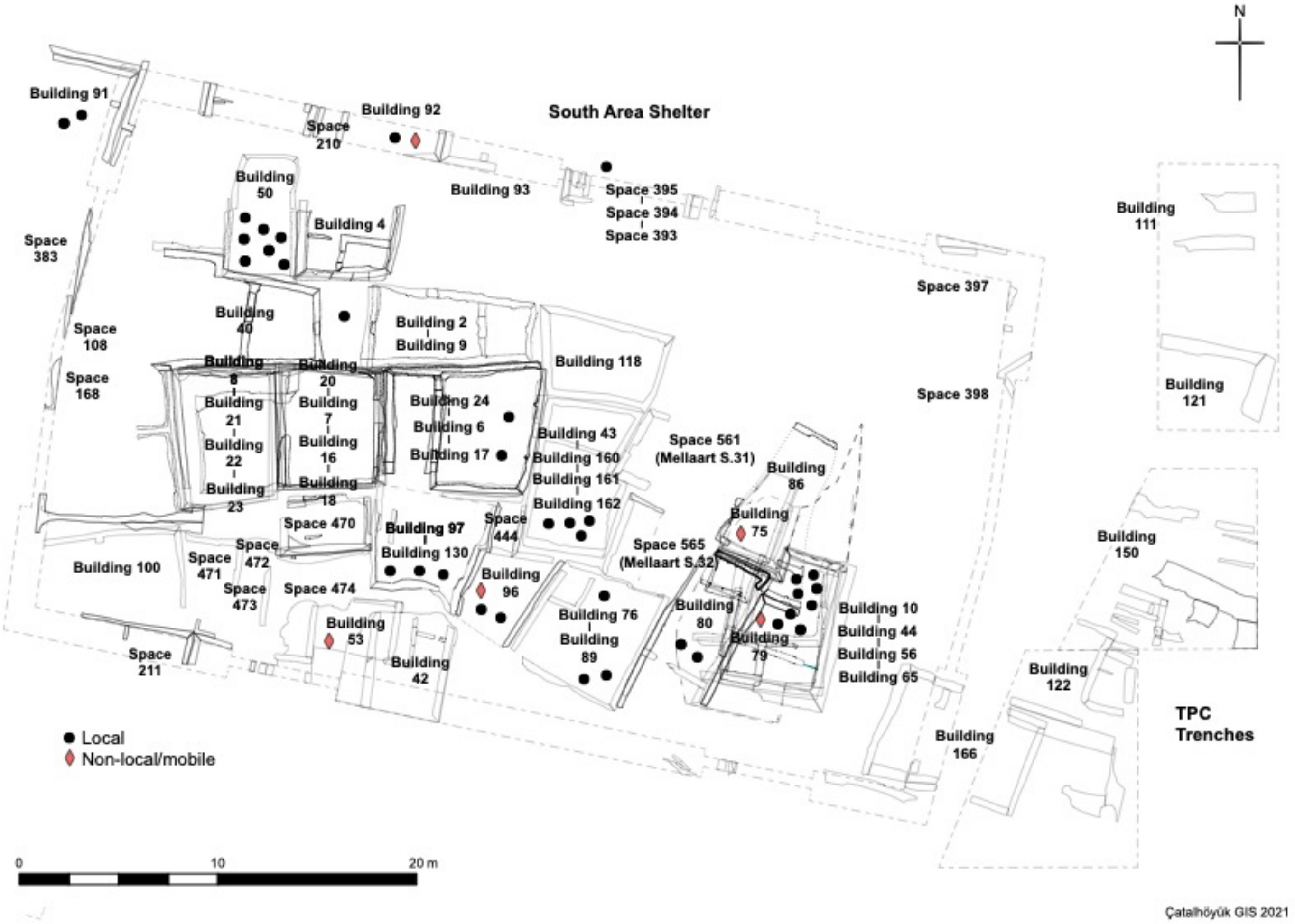

SI Table S1: Numbers of samples and interquartile range (IQR) for Boncuklu and Çatalhöyük human enamel phosphate.

**$\delta^{18}O_p$**	**N**	**IQR (Lower)**	**IQR (Upper)**
Çatalhöyük	77	15.49	18.67
Boncuklu	18	13.57	20.24

SI Table S2: Expected Phosphate $\delta^{18}O_{(VSMOW)}$ values for Central Turkey humans calculated from local meteoric waters reported in Henton et al. (31) using formulae cited in Pollard et al. (2011). *formula represents a "superset" or combination of these formulae.

Reference	Equation	Mean range (-7‰ to -9‰)	Weighted annual mean (-8.4‰)	Min (Winter) value (-12‰)	Max (Summer) Value (-4‰)
Luz et al. (43)	y= 0.752x + 22.00	16.74 – 15.23	15.68	12.98	18.99
Longinelli et al. (44)	y= 0.633x + 22.29	17.86 – 16.59	16.97	14.69	19.76
Levinson et al. (45)	y= 0.470x + 19.23	15.94 – 15.00	15.28	13.59	17.35
Daux et al. (46)	y= 0.501x + 20.71	17.20 – 16.20	16.50	14.70	18.71
*Pollard et al. (47)	y= 0.531x + 20.52	16.80 – 15.74	16.06	14.15	18.40

SI Table S3: Raw Sr and O data (Non-local/more mobile individuals in bold italic) M = Male, M? = possible male, X = indeterminate, F? = possible female, F = female

Site	Context	87Sr/86Sr	d18OCarbVSMOW	d18Opo4VSMOW	Sex	Age
Pınarbaşı	PB14 ZBD	0.70789	27.20	18.39	M	Adult
Pınarbaşı	PB9 ABM	0.70779	24.47	15.57	X	Subadult
Pınarbaşı	PB9 ZAN	0.70789	24.07	15.16	F?	Adult
Pınarbaşı	PB9 ZAQ	0.70791	23.66	14.74	X	Adult
Boncuklu	BK ZHAF	0.70794	24.94	16.06	F	Adult
Boncuklu	BK ZHAJ	0.70794	25.31	16.44	F	Adult
Boncuklu	***BK ZHB***	***0.70803***	***29.23***	***20.49***	***F***	***Adult***
Boncuklu	BK ZHM	0.70796	26.68	17.85	F	Adult
Boncuklu	BK ZQM	0.70801	26.36	17.52	F?	Adult
Boncuklu	BK ZHBD	0.70797	25.15	16.27	M	Adult
Boncuklu	BK ZHBJ	0.70799	24.96	16.08	M	Adult
Boncuklu	BK ZHF	0.70792	26.61	17.78	M	Adult
Boncuklu	BK ZHJ	0.70796	26.62	17.79	M	Adult
Boncuklu	BK ZKO	0.70801	26.20	17.36	M	Adult
Boncuklu	BK ZMM	0.70799	24.74	15.85	M	Adult
Boncuklu	BK ZMOJ	0.70796	23.97	15.06	M	Adult
Boncuklu	BK ZQC	0.70800	25.90	17.05	M?	Adult
Boncuklu	BK MMBF	0.70793	24.63	15.74	X	Adult
Boncuklu	BK ZHP	0.70785	26.43	17.60	X	Subadult
Boncuklu	BK ZMNN	0.70787	26.72	17.90	X	Adult
Boncuklu	BK ZMNR	0.70787	23.52	14.59	X	Adult
Boncuklu	BK ZQI	0.70805	26.17	17.33	X	Subadult
Çatalhöyük	CH 01926	0.70801	26.42	17.59	F	Adult
Çatalhöyük	CH 01949	0.70801	26.37	17.53	F	Adult
Çatalhöyük	CH 02115	0.70799	25.79	16.94	F	Adult
Çatalhöyük	CH 02529	0.70797	25.74	16.88	F	Adult
Çatalhöyük	CH 05169	0.70769	23.55	14.62	F	Adult
Çatalhöyük	CH 05818	0.70793	25.29	16.42	F	Adult
Çatalhöyük	CH 08115	0.70799	26.10	17.26	F	Adult
Çatalhöyük	CH 11649	0.70796	25.78	16.93	F	Adult
Çatalhöyük	CH 11665	0.70794	26.49	17.66	F	Adult
Çatalhöyük	CH 13124	0.70802	26.18	17.34	F	Adult

SI Table S3: Raw Sr and O data (continued)

Site	Context	87Sr/86Sr	d18OCarbVSMOW	d18Opo4VSMOW	Sex	Age
Çatalhöyük	CH 13609	0.70795	25.10	16.22	F	Adult
Çatalhöyük	***CH 14032***	***0.70820***	***24.73***	***15.84***	***F***	***Adult***
Çatalhöyük	CH 14441	0.70797	26.17	17.33	F	Adult
Çatalhöyük	***CH 14753***	***0.70706***	***25.04***	***16.16***	***F***	***Adult***
Çatalhöyük	CH 19038	0.70802	26.03	17.18	F	Adult
Çatalhöyük	CH 19224	0.70794	26.17	17.33	F	Adult
Çatalhöyük	***CH 20824***	***0.70818***	***26.53***	***17.70***	***F***	***Adult***
Çatalhöyük	CH 21550	0.70797	25.81	16.96	F	Adult
Çatalhöyük	CH 21571	0.70802	25.57	16.71	F	Adult
Çatalhöyük	CH 21672	0.70794	25.39	16.52	F	Adult
Çatalhöyük	CH10829	0.70790	26.67	17.84	F	Adult
Çatalhöyük	CH 01481	0.70802	27.39	18.59	F?	Adult
Çatalhöyük	CH 01995	0.70794	24.91	16.03	F?	Adult
Çatalhöyük	CH 02058	0.70795	25.77	16.91	F?	Adult
Çatalhöyük	CH 05750	0.70795	25.43	16.56	F?	Adult
Çatalhöyük	***CH 05774***	***0.70854***	***24.62***	***15.73***	***F?***	***Adult***
Çatalhöyük	CH 12863	0.70786	25.81	16.96	F?	Adult
Çatalhöyük	CH 13125	0.70795	25.80	16.95	F?	Adult
Çatalhöyük	CH 13132	0.70796	26.16	17.32	F?	Adult
Çatalhöyük	***CH 16411***	***0.70981***	***26.62***	***17.79***	***F?***	***Adult***
Çatalhöyük	CH 20351	0.70795	25.70	16.84	F?	Adult
Çatalhöyük	CH 20377	0.70798	26.46	17.63	F?	Adult
Çatalhöyük	CH 20830	0.70801	26.07	17.22	F?	Adult
Çatalhöyük	CH 21685	0.70795	26.24	17.40	F?	Adult
Çatalhöyük	CH 30351	0.70792	25.89	17.04	F?	Adult
Çatalhöyük	CH 30928	0.70797	25.52	16.66	F?	Adult
Çatalhöyük	CH 08598	0.70790	26.00	17.15	M	Adult
Çatalhöyük	CH 30007	0.70799	26.82	18.00	M	Adult
Çatalhöyük	CH 01924	0.70794	25.27	16.40	M	Adult
Çatalhöyük	CH 02056	0.70790	25.11	16.23	M	Adult
Çatalhöyük	CH 02169	0.70800	25.81	16.96	M	Adult
Çatalhöyük	CH 03368	0.70801	25.28	16.41	M	Adult
Çatalhöyük	CH 10813	0.70807	25.55	16.69	M	Adult
Çatalhöyük	CH 10840	0.70797	25.82	16.97	M	Adult

SI Table S3: Raw Sr and O data (continued)

Site	Context	87Sr/86Sr	d18OCarbVSMOW	d18Opo4VSMOW	Sex	Age
Çatalhöyük	CH 12875	0.70777	25.06	16.18	M	Adult
Çatalhöyük	CH 13126	0.70798	26.02	17.17	M	Adult
Çatalhöyük	CH 13133	0.70802	25.79	16.94	M	Adult
Çatalhöyük	CH 14507	0.70797	25.93	17.08	M	Adult
Çatalhöyük	***CH 16513***	***0.70903***	***28.14***	***19.36***	***M***	***Adult***
Çatalhöyük	CH 17485	0.70802	26.42	17.59	M	Adult
Çatalhöyük	CH 02886	0.70796	25.72	16.86	M?	Adult
Çatalhöyük	CH 04394	0.70793	25.94	17.09	M?	Adult
Çatalhöyük	CH 05658	0.70796	25.45	16.58	M?	Adult
Çatalhöyük	CH 08113	0.70799	25.89	17.04	M?	Adult
Çatalhöyük	CH 11493	0.70791	26.40	17.57	M?	Adult
Çatalhöyük	CH 11647	0.70783	26.86	18.04	M?	Adult
Çatalhöyük	CH 11655	0.70795	25.58	16.72	M?	Adult
Çatalhöyük	CH 11739	0.70798	26.68	17.85	M?	Adult
Çatalhöyük	***CH 14805***	***0.70871***	***no data***	***no data***	***M?***	***Adult***
Çatalhöyük	CH 20810	0.70797	24.96	16.08	M?	Adult
Çatalhöyük	CH 01425	0.70802	26.31	17.47	X	Adult
Çatalhöyük	CH 01884	0.70793	25.88	17.03	X	Adult
Çatalhöyük	CH 01922	0.70802	25.02	16.14	X	Adult
Çatalhöyük	CH 01925	0.70802	25.71	16.85	X	Adult
Çatalhöyük	CH 08114	0.70799	26.17	17.33	X	Adult
Çatalhöyük	CH 08409	0.70803	26.28	17.44	X	Adult
Çatalhöyük	CH 08423	0.70802	26.02	17.17	X	Adult
Çatalhöyük	CH 10529	0.70801	26.36	17.52	X	Adult
Çatalhöyük	CH 11982	0.70799	26.35	17.51	X	Adult
Çatalhöyük	CH 12935	0.70799	25.73	16.87	X	Adult
Çatalhöyük	CH 16601	0.70802	26.32	17.48	X	Adult
Çatalhöyük	CH 16638	0.70799	25.78	16.93	X	Adult
Çatalhöyük	CH 18447	0.70797	25.12	16.24	X	Adult
Çatalhöyük	CH 18464	0.70796	24.74	15.85	X	Adult
Çatalhöyük	CH 21700	0.70796	26.31	17.47	X	Adult
Çatalhöyük	CH 21778	0.70796	26.45	17.62	X	Adult
Çatalhöyük	CH 21802	0.70797	26.53	17.70	X	Adult

SI References

1. M. Feldman *et al.*, Late Pleistocene human genome suggests a local origin for the first farmers of central Anatolia. *Nat. Commun.* 10.1038/s41467-019-09209-7 (2019).
2. D. Baird *et al.*, Juniper smoke, skulls and wolves' tails. The Epipalaeolithic of the Anatolian plateau in its South-west Asian context; insights from Pınarbaşı. *Levant.* **45**, 175–209 (2013).
3. D. Baird *et al.*, Agricultural Origins on the Anatolian Plateau. *Proc. Natl. Acad. Sci. U.S.A.* **115**, E3077–E3086 (2018).
4. E. Baysal, A tale of two assemblages: early Neolithic manufacture and use of beads in the Konya Plain. *Anatol. Stud*. **63**, 1–16 (2013).
5. D. Baird, A. Fairbairn, L. Martin, The Animate House: The Institutionalization of the household in Neolithic Central Anatolia. *World Archaeol.* **49**, 753–776 (2016).
6. I. Hodder, "Changing Çatalhöyük Worlds" in *Peopling the Landscape of Çatalhöyük*. I. Hodder, Ed. (British Institute at Ankara, 2021). pp. 1–29.
7. C. Nakamura, L. Meskell, Articulate Bodies: Forms and Figures at Çatalhöyük. *J. Archaeol. Method Theory*.**16**, 205–230 (2009).
8. K. C. Twiss, J. Wolfhagen, G. A. Demirergi, J. A. Mulville, "Macromammals of Çatalhöyük: new practices and durable traditions" in *Peopling the Landscape of Çatalhöyük: Reports from the 2009-2017 Seasons*, I. Hodder, Ed. (British Institute at Ankara, 2021), pp. 145–180.
9. A. Bogaard A *et al.*, "The archaeobotany of Çatalhöyük: results from 2009–2017 excavations and final synthesis" in *Peopling the Landscape of Çatalhöyük: Reports from the 2009-2017 Seasons*, I. Hodder, Ed. (British Institute at Ankara, 2021), pp. 91–124.
10. J.-P. Bocquet-Appel & O Bar-Yosef, Eds. *The Neolithic Demographic Transition and its Consequences* (Springer, 2008).
11. I. Kuijt, People and Space in Early Agricultural Villages: Exploring Daily Lives, Community Size, and Architecture in the Late Pre-Pottery Neolithic. *J. Anthropol. Archaeol.* **19**, 75–102 (2000).
12. C. S. Larsen *et al.*, Bioarchaeology of Neolithic Çatalhöyük reveals fundamental transitions in health, mobility, and lifestyle in early farmers. *Proc. Natl. Acad. Sci. U.S.A.* **116**, 12615–12623 (2019).
13. E. B. Banning, B. F. Byrd. Houses and the Changing Residential Unit: Domestic Architecture at PPNB 'Ain Ghazal, Jordan. *Proc. Prehist.Soc.* **53**, 309–325 (1987).
14. K. Croucher *Death and Dying in the Neolithic Near East* (Oxford University Press, 2012)
15. K. V. Flannery, The origins of the village revisited: from nuclear to extended households. *Am. Antiq.* **67**, 417–433 (2002).
16. Hodder, P. Pels, "History Houses" in *Religion in the Emergence of Civilization: Çatalhöyük as a Case Study*, I. Hodder, Ed. (Cambridge University Press, 2010), pp. 163–186.
17. T. I. Molleson, "Appendix 5: the human remains" in *Village on the Euphrates: The Excavation of Abu Hureyra*, A.M.T. Moore, G.C. Hillman, A.J. Legge, Eds. (Oxford University Press, 2000), pp. 533-544.
18. T. I. Molleson, J. Ottevanger, T. Compton, Variations in Neolithic Teeth from Çatalhöyük. *Anatol. Stud.* **54**, 1–26 (2004).
19. M. Pilloud, C. S. Larsen, "Official" and "Practical" Kin: Inferring Social and Community Structure From Dental Phenotype at Neolithic Çatalhöyük, Turkey. *Am. J. Phys. Anthropol.* **145**, 519–530 (2011).
20. K. W. Alt, M. Benz, W. Vach, T. L. Simmons, N. Goring-Morris, Insights into the Social Structure of the PPNB Site of Kfar HaHoresh, Israel, Based on Dental Remains. *PLoS One*, 10.1371/journal.pone.0134528 (2015).
21. 23. K. W. Alt *et al.*, Earliest Evidence for Social Endogamy in the 9,000-Year-Old-Population of Basta, Jordan. *PLoS One* 10.1371/journal.pone.0065649 (2013).

22. J. Santana *et al.*, Multi-isotope evidence of population aggregation in the Natufian and scant migration during the early Neolithic of the Southern Levant. *Sci. Rep.* 10.1038/s41598-021-90795-2 (2021).
23. I. Lazaridis *et al.*, Genomic Insights into the origin of farming in the ancient Near East. *Nature.* **536**, 419–426 (2016).
24. G. M. Kılınç *et al.*, Archaeogenomic analysis of the first steps of Neolithization in Anatolia and the Aegean. *Proc. Biol. Sci.,* 10.1098/rspb.2017.2064 (2017).
25. T. Carter, G. Poupeau, C. Bressy, N. J. G. Pearce "From Chemistry to Consumption: Towards a History of Obsidian Use at Çatalhöyük through a programme of Inter-laboratory Trace-elemental Characterisation" in *Changing Materialities at Çatalhöyük: Reports from the 1995-1999 Seasons*, I. Hodder, Ed. (McDonald Institute of Archaeology, 2005), pp. 285–306.
26. M. Chyleński *et al.*, Ancient Mitochondrial Genomes Reveal the Absence of Maternal Kinship in the Burials of Çatalhöyük People and their Genetic Affinities. *Genes (Basel)*. 10.3390/genes10030207 (2019).
27. R. Yaka *et al.*, Variable Kinship Patterns in Neolithic Anatolia Revealed by Ancient Genomes. *Curr. Biol.* **31**, 1–14 (2021).
28. H. Ringbauer, J. Novembre, M. Steinrücken, Human Parental Relatedness through Time - Detecting Runs of Homozygosity in Ancient DNA. *Nat. Commun.* **12**, 5425 (2021).
29. A. Bogaard, *et al.*, Locating Land Use at Neolithic Çatalhöyük, Turkey: The Implications of $^{87}Sr/^{86}Sr$ signatures in Plants and Sheep Tooth Sequences. *Archaeometry.* **56**, 860–877 (2014).
30. J. A. Evans, C. A. Chenery, J. Montgomery, A summary of strontium and oxygen isotope variation in archaeological human tooth enamel excavated from Britain. *J. Anal. At. Spectrom.* **27**, 754–764 (2012).
31. E. Henton, W. Meier-Augenstein, H. F. Kemp, The Use of Oxygen Isotopes in Sheep Molars to Investigate Past Herding Practices at The Neolithic Settlement of Çatalhöyük, Central Anatolia. *Archaeometry.* **52**, 429–449 (2010).
32. R. A. Bentley, T. D. Price, E. Stephan, Determining the "local" $^{87}Sr/^{86}Sr$ range for archaeological skeletons: a case study from Neolithic Europe. *J Archaeol Sci*. **31**, 365–375 (2004).
33. G. Faure, T. M. Mensing, T.M, *Isotopes: principles and applications* (Wiley, 2005).
34. R. A. Bentley, Strontium isotopes from the earth to the archaeological skeleton: a review. *J. Archaeol. Method Theory.* **13**, 135–187 (2006).
35. E. Lightfoot, T. C. O'Connell, On the use of biomineral oxygen isotope data to identify human migrants in the archaeological record: Sample variation, statistical methods and geographical considerations. *PLoS One*. 10.1371/journal.pone.0153850 (2016).
36. R. Brettell, J. Montgomery, J. Evans, Brewing and stewing: the effect of culturally mediated behaviour on the oxygen isotope composition of ingested fluids and the implications for human provenance studies. *J. Anal. At. Spectrom.* **27**, 778–785 (2012).
37. S. B. Roberts et al., Effect of weaning on accuracy of doubly labeled water method in infants. *Am. J. Physiol.* **254**, R622–R627 (1988).
38. S. J. AlQahatani, H. M. Liversidge, M. P. Hector, M.P. Atlas of tooth development and eruption *Am. J. Phys. Anthropol.* **142**, 481–90 (2010).
39. T. B. Coplen, Normalization of oxygen and hydrogen isotope data. *Chem. Geol.* **72**, 293–297 (1988).
40. C. A. Chenery, V. Pashley, A. L. Lamb, H. J. Sloane, J. A. Evans. The oxygen isotope relationship between the phosphate and structural carbonate fractions of human bioapatite. *Rapid Commun. Mass Spectrom.* **26**, 309–319 (2012).
41. N. Roberts, O. Erol, T. de Meester, H.-P. Uerpmann. Radiocarbon chronology of late Pleistocene Konya lake. *Nature*. **281**, 662–664 (1979).
42. J. L. Birck, Precision K–Rb–Sr isotopic analysis—application to Rb–Sr chronology *Chem. Geol.* **56**, 73–83 (1986).

43. N. Roberts, A. Rosen, Diversity and Complexity in Early Farming Communities of Southwest Asia: New Insights into the Economic and Environmental Basis of Neolithic Çatalhöyük. *Curr. Anthropol.* **50**, 393–402 (2009).
44. B. Luz, Y. Kolodny, M. Horowitz M, Fractionation of oxygen isotopes between mammalian bone-phosphate and environmental drinking water. *Geochim. Cosmochim. Acta*. **48**,1689–1693 (1984).
45. A. Longinelli, Oxygen isotopes in mammal bone phosphate: a new tool for paleohydrological and paleoclimatological research? *Geochim. Cosmochim. Acta* **48**, 385–390 (1984).
46. A. A. Levinson, B. Luz, Y. Kolodny, Variations in oxygen isotopic compositions of human teeth and urinary stones. *Appl. Geochem.* **2**, 367–371 (1987).
47. V. Daux, *et al*., Oxygen isotope fractionation between human phosphate and water revised. *J Hum Evol.* **55**, 1138–1147 (2008).
48. A. M. Pollard, M. Pellegrini, J. A. Lee Thorp, Technical Note: Some Observations on the Conversion of Dental Enamel d18Op Values to d18Ow to Determine Human Mobility. *Am. J. Phys. Anthropol.* **145**, 499–504 (2011).
